# What is the better choice for nurses? Alternating air pressure mattresses versus static air mattresses to prevent pressure ulcers in elderly hospitalized patients: A protocol for systematic review and meta-analysis

**DOI:** 10.1097/MD.0000000000029084

**Published:** 2022-04-01

**Authors:** Yinxi Li, Xuemei Zeng, Jianyuan Wang, Chunlei Wang

**Affiliations:** a Dermatological Department, Wuhan Xinzhou District People's Hospital, Wuhan, China; b Nursing Department, Wuhan Xinzhou District People's Hospital, Wuhan, China; c Orthopedics Department, Wuhan Xinzhou District People's Hospital, Wuhan, China.

**Keywords:** alternating air pressure mattresses, meta-analysis, pressure ulcers, protocol, static air mattresses, systematic review

## Abstract

**Background:**

Evidence on the comparative effectiveness between commercially available support surfaces in preventing pressure ulcer development is lacking. The purpose of this study was to compare the efficacy and safety of alternating air pressure mattresses (AAPMs) versus static air mattresses to prevent pressure ulcers in elderly hospitalized patients and to provide evidence for clinical practice.

**Methods:**

The electronic databases of Cochrane Library, EMBASE, PubMed, and Web of Science will be searched in April 2022 using the following key terms: “pressure ulcers,” “support surface,” and “pressure mattresses,” for all relevant studies. Only English publications are included. The primary outcome is the incidence of pressure ulcers; secondary outcomes include patient satisfaction, cost, and other bedridden complications. The Cochrane risk of bias tool will be independently used to evaluate the risk of bias of included randomized cohort studies by 2 reviewers. A modified version of the Downs and Black tool is adopted to evaluate the quality of nonrandomized cohort studies. All outcomes are pooled on random-effect model.

**Results:**

We hypothesized that group with AAPMs will provide better therapeutic benefits compared with control group.

**Conclusions:**

It is worthy to critically review the evidence of the assessment of AAPMs and static air mattresses to inform clinical practice.

**OSF registration number:**

10.17605/OSF.IO/MYPZ2.

## 1 Introduction

Pressure ulcers (PUs) are one of the most common complications of bedridden patients and are caused by unrelieved pressure and shear forces. These 2 forces can interrupt blood circulation to the underlying tissues, resulting in a lack of oxygen to the soft tissues and muscles.^[1]^ Advanced age was identified as a predictive risk factor for PUs, and the cumulative presence of risk factors placed older adults at high risk. PUs not only increase the morbidity of elderly and infirm patients, but also increase mortality. They can cause pain and discomfort, seriously impair quality of life, and increase health costs.^[2,3]^ Literature reviews between January 2000 and December 2012 showed that the prevalence of pressure ulcers in aged care facilities ranged from 4.1% to 32.2%, with an incidence ranging from 1.9% to 59%.^[4–6]^

International guidelines recommend the use of a decompression support surface for all at-risk patients. Currently, there are a variety of commercially available reduced-pressure support surfaces, such as alternating air pressure mattresses (AAPMs) and static air mattresses (SAMs).^[7]^ AAPMs achieve pressure redistribution through cyclic inflation and deflation of the air cells, regardless of whether the patient's weight is on the surface. Pressure redistribution can allow the patient to sink into the mattress, thereby increasing the contact area between the patient and the support surface.^[8,9]^ SAMs are always overlay mattress. The mattress overlay is compact and low in weight. It consists of several compartments; the air moves over a large area when a person lies on the mattress.^[9–11]^

Unfortunately, evidence on the comparative effectiveness between commercially available support surfaces in preventing PUs development is lacking. Selecting the most appropriate support surface for each individual patient involves various factors and complexities. The decision to use a reduced-pressure support surface is based on individual characteristics, such as the results of a risk assessment, patient comfort, general health, training, and material availability and resources. It is worthy to critically review the evidence of the assessment of AAPMs and SAMs to inform clinical practice. Therefore, the purpose of this study was to compare the efficacy and safety of AAPMs versus SAMs to prevent PUs in elderly hospitalized patients and to provide evidence for clinical practice.

## 2 Materials and methods

### 2.1 Selection of studies

The systematic review protocol has been registered on Open Science Framework registries. Two independent investigators followed The Preferred Reporting Items for Systematic Reviews and Meta-Analyses reporting guidelines and the recommendations of the Cochrane Collaboration to conduct this meta-analysis. The electronic databases of Cochrane Library, EMBASE, PubMed, and Web of Science will be searched in April 2022 using the following key terms: “pressure ulcers,” “support surface,” and “pressure mattresses,” for all relevant studies. Additionally, the reference lists from published original articles and relevant reviews are assessed to identify more relevant studies. Only English publications are included. Ethical approval is not necessary because the present meta-analysis will be performed on the basis of previous published studies.

### 2.2 Inclusion and exclusion criteria

Study included in this systematic review and meta-analysis has to meet all of the following inclusion criteria:

Population: elderly hospitalized patients requiring prolonged bed rest without PUs;
Intervention: group with AAPMs;
Comparator: group with SAMs;
Outcomes: the primary outcome is the incidence of PUs; secondary outcomes include patient satisfaction, cost, and other bedridden complications.
Study design: cohort studies
The exclusion criteria are as follows: studies which do not assessed the above outcomes; no direct comparison of AAPMs and SAMs; studies with the following types: case reports, comments or letters, biochemical trials, protocols, conference abstracts, and reviews.

### 2.3 Data extraction

Two authors will conduct data extraction independently, and when disagreements persist, a third author will be consulted. Reviewers are not blinded to information about authors, journals, or the results of each reviewed article. Extracted data on participants, type of intervention, duration of follow-up, clinical outcome measures, and reported outcomes will be processed using standardized tables. The table used is detailed according to the Cochrane Handbook for Systematic Reviews of Interventions-Version 5.1.0.

### 2.4 Risk of bias assessment

The Cochrane risk of bias tool will be independently used to evaluate the risk of bias of included randomized cohort studies by 2 reviewers. The quality will be assessed by using following 7 items: random sequence generation, allocation concealment, blinding of participants and personnel, blinding of outcome assessment, incomplete outcome data, selective reporting, and other bias. A modified version of the Downs and Black tool is adopted to evaluate the quality of nonrandomized cohort studies. The modified version consists of 27 items with a total possible score of 29. A score of ≥75% indicates high quality, 60% to 74% indicates moderate quality, and ≤60% low quality. Two investigators independently evaluate included studies on the 27 criteria, with any discrepancies resolved by a third independent reviewer.

### 2.5 Data synthesis

The present study will be performed by Review Manager Software (RevMan Version 5.3, The Cochrane Collaboration, Copenhagen, Denmark). We use the Mantel–Haenzel method to calculate the pooled odds ratio. Odds ratio with a 95% confidence interval is assessed for dichotomous outcomes. *P* < .05 is set as the significance level. The heterogeneity is assessed by using the *Q* test and *I*^2^ statistic. When *I*^2^ ≥ 40%, it is considered to represent significant heterogeneity. All outcomes are pooled on random-effect model. The Z test is used to assess the overall effect. The publication bias will be assessed by using funnel plots diagram. The funnel plot asymmetry will be evaluated by an Egger linear regression test to reveal any possible publication bias. Sensitivity analyses will be undertaken to determine the potential source of heterogeneity when significant.

## 3 Discussion

The presence of PUs is internationally accepted as an important indicator of the quality of care. Unfortunately, evidence on the comparative effectiveness between commercially available support surfaces in preventing PUs development is lacking. Selecting the most appropriate support surface for each individual patient involves various factors and complexities. The decision to use a reduced-pressure support surface is based on individual characteristics, such as the results of a risk assessment, patient comfort, general health, training and material availability, and resources. It is worthy to critically review the evidence of the assessment of AAPMs and SAMs to inform clinical practice. Therefore, the purpose of this study was to compare the efficacy and safety of AAPMs versus SAMs to prevent PUs in elderly hospitalized patients and to provide evidence for clinical practice.

## Author contributions

**Conceptualization:** Chunlei Wang, Jianyuan Wang.

**Data curation:** Yinxi Li, Xuemei Zeng.

**Formal analysis:** Yinxi Li, Xuemei Zeng.

**Funding acquisition:** Jianyuan Wang.

**Investigation:** Yinxi Li, Xuemei Zeng.

**Methodology:** Chunlei Wang.

**Supervision:** Jianyuan Wang, Chunlei Wang.

**Writing – original draft:** Yinxi Li, Xuemei Zeng.

**Writing – review & editing:** Jianyuan Wang.
